# Normalization of Voltage-Sensitive Dye Signal with Functional Activity Measures

**DOI:** 10.1371/journal.pone.0004041

**Published:** 2008-12-24

**Authors:** Kentaroh Takagaki, Michael Thomas Lippert, Benjamin Dann, Tim Wanger, Frank W. Ohl

**Affiliations:** 1 Leibniz Institute for Neurobiology, Magdeburg, Germany; 2 School of Medicine, Georgetown University, Washington, D. C., United States of America; 3 Max Planck Institute for Biological Cybernetics, Tübingen, Germany; 4 Max Planck Institute for Brain Research, Frankfurt/Main, Germany; 5 Institute of Biology, Otto-von-Guericke-University, Magdeburg, Germany; Vrije Universiteit Amsterdam, Netherlands

## Abstract

In general, signal amplitude in optical imaging is normalized using the well-established ΔF/F method, where functional activity is divided by the total fluorescent light flux. This measure is used both directly, as a measure of population activity, and indirectly, to quantify spatial and spatiotemporal activity patterns. Despite its ubiquitous use, the stability and accuracy of this measure has not been validated for voltage-sensitive dye imaging of mammalian neocortex *in vivo*. In this report, we find that this normalization can introduce dynamic biases. In particular, the ΔF/F is influenced by dye staining quality, and the ratio is also unstable over the course of experiments. As methods to record and analyze optical imaging signals become more precise, such biases can have an increasingly pernicious impact on the accuracy of findings, especially in the comparison of cytoarchitechtonic areas, in area-of-activation measurements, and in plasticity or developmental experiments. These dynamic biases of the ΔF/F method may, to an extent, be mitigated by a novel method of normalization, ΔF/ΔF_epileptiform_. This normalization uses as a reference the measured activity of epileptiform spikes elicited by global disinhibition with bicuculline methiodide. Since this normalization is based on a functional measure, i.e. the signal amplitude of “hypersynchronized” bursts of activity in the cortical network, it is less influenced by staining of non-functional elements. We demonstrate that such a functional measure can better represent the amplitude of population mass action, and discuss alternative functional normalizations based on the amplitude of synchronized spontaneous sleep-like activity. These findings demonstrate that the traditional ΔF/F normalization of voltage-sensitive dye signals can introduce pernicious inaccuracies in the quantification of neural population activity. They further suggest that normalization-independent metrics such as waveform propagation patterns, oscillations in single detectors, and phase relationships between detector pairs may better capture the biological information which is obtained by high-sensitivity imaging.

## Introduction

Voltage-sensitive dye imaging (VSDI) is the best-suited method for imaging fast propagation of coherent population activity in the neocortex [1] and other excitable tissues [2], [3], and in patterned growth cardiac myocyte networks in culture [4], [5]. Recent advances in dye chemistry [6] and the maturation of measuring apparatus [7] now allow routine imaging of hundreds of trials of such activity with high sensitivity, without averaging [1], [8], [9].

When analyzing such data, previous studies have mainly quantified spatiotemporal patterns [10], [11], [12], global spatial metrics such as area of activation and point-spread function [11], [13], or latency [12], [14], [15]. However, high-sensitivity imaging also allows novel methods of analysis which do not rely on signal amplitude, per se [16].

High-sensitivity imaging should also allow accurate quantification of the amplitude of cortical mass action, both within the experimental field of view, and between experiments. For instance, one recurring scientific question in voltage-sensitive dye imaging is the nature of the interaction between “spontaneous” population activity and evoked population activity [9], [17]. While these pioneering studies have reported such interaction in very stark qualitative terms, advances in high-sensitivity imaging should allow refinement of these principles based on strict statistical tests of information theory. However, although previous measurements have high precision, it is difficult to obtain accuracy, which would be necessary to compare between experiments and experimental conditions. We have embarked on a program to quantify the amplitude effects of such spontaneous-evoked interactions both within and across sensory modalities, under varying states of the cortical network. As a first step in this program, we have reevaluated the quantification of the amplitude of neural mass action, as recorded in voltage-sensitive dye imaging, in order to ensure both the accuracy and precision of our measurements.

In this report, we provide evidence to demonstrate that the classical ΔF/F method of normalizing functional signal can introduce dynamically-changing biases in amplitude quantification of neural activity. This finding raises questions regarding both the direct use of the ΔF/F measure, and regarding the use of derivative measures such as area of activation. Furthermore, both these biases and their time courses are influenced by the quality of dye staining, which is area dependent. Therefore, this bias is potentially of great concern when comparing between cortical areas, and when comparing across time in plasticity paradigms. We provide evidence to suggest that this bias is largely due to staining of non-neuronal elements within the cortical mantle, which will increase the total fluorescent flux (F), but will not affect the functional signal (ΔF) to an equal or proportional extent. To illuminate this problem, we describe a novel method of normalization, ΔF/ΔF_epileptiform_, which uses a functional basis of normalization to obtain a more robust amplitude measure. We provide evidence to suggest that such a measure can better represent the functional amplitude of population mass action. Since such pseudo-epileptic states are not practical for many physiological experiments, we also discuss alternative functional normalizations based on the amplitude of synchronized spontaneous sleep-like activity.

## Materials and Methods

### Surgery

Experiments were conducted with 19 adult male Wistar rats (250–400 g) in accordance with authorizations approved by the Ethics Committee of the State of Sachsen-Anhalt, Germany (42502-2-825). For detailed procedures, see our previous work [8]. Briefly, animals were anesthetized with urethane (1.25 g/kg, IP), or isoflurane where noted. Animals were monitored to ensure sufficient anesthetic plane, and anesthesia was augmented with supplemental doses of urethane or xylazine as necessary. Physiological support included maintenance of normothermia and maintenance of corneal hydration with a bland ophthalmic ointment. Cranial windows were drilled over visual cortex (bregma −4 to −9 mm, lateral 1 to 6 mm). Particular care was taken to avoid heat and pressure trauma from the drill tip.

### Voltage-Sensitive Dye Imaging

Staining procedures are described in detail in [8]. Briefly, the dural surface was washed with Ringer solution (103 mM NaCl, 5.5 mM KCl, 1.7 mM CaCl_2_2H_2_O, 28 mM sodium lactate) or ACSF (124 mM NaCl, 4.9 mM KCl, 1.2 mM KH_2_PO_4_, 2.0 mM MgSO_4_, 2.0 mM CaCl2, 24.6 mM NaHCO3, and 10 mM D-glucose), to remove any residual blood. After drying the dura thoroughly to improve permeability [8], voltage-sensitive dye RH-1691 (2 mg/ml; Optical Imaging, Rehovoth, Israel) desolved at 2 mg/ml in Ringer or ACSF solution was applied transdurally with constant circulation, for 1 to 1.5 hrs. Unless otherwise noted, the staining solution contained 1 mg/ml of bicuculline methiodide, thereby inducing a stable pseudoepileptic state [18], [19]. After staining, excess dye was washed off with dye-free solution for >15 min. Where noted in the text, a cisternal puncture was performed to drain up to 100 µl of cerebrospinal fluid, and durotomy was performed.

**Figure 1:**
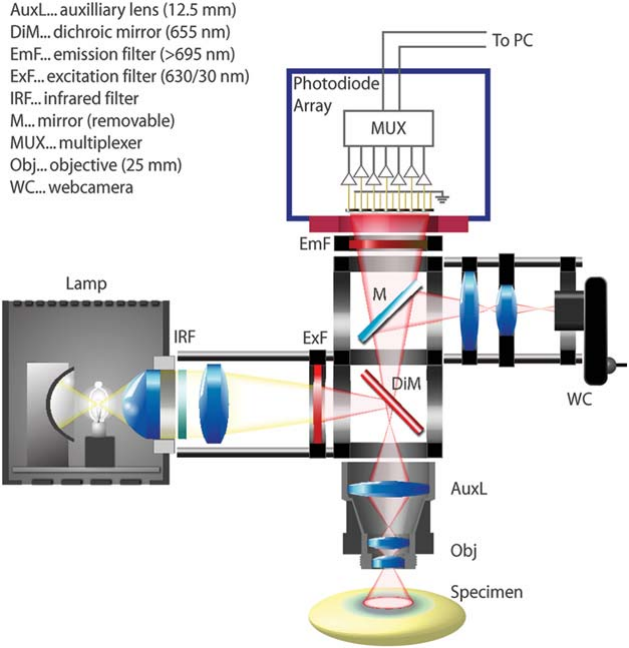
Schematic of the voltage-sensitive dye imaging apparatus. Affordable optical components can be assembled with standard mountings to build a macroscope with high numerical aperture. The use of large numerical aperture optics and high-quality filters allows recording with high signal-to-noise ratio in single trials. Since the photodiode array itself has insufficient spatial resolution for micrography and focusing, an equifocal web camera is also incorporated in the setup.

The recording apparatus is modified and improved from that described previously [Figure 1]. The macroscope was custom-designed to obtain a large numerical aperture while minimizing light path [8], [20]. The main component is a 25 mm video camera lens with NA of 0.45 (DO-2595, Navitar, USA), supplemented with a 100 mm lens to provide a total focal distance of 20 mm (Figure 1). Illumination utilized a 100 W Tungsten-Halogen microscope lamp (HAL100, Carl Zeiss, Germany) powered by a stabilized power source (PS2316-050, EA Elektro-Automatik, Germany). This light was filtered at 630±15 nm (BK Interferenz Optik, Germany), and reflected onto the cortex via a 655 nm dichroic mirror (Omega Optical, USA). The illumination was adjusted to approximate Köhler illumination 200 µm below the cortical surface. Light from the sample was passed through a 695 nm long pass filter and collimated to the imaging aperture of a 464-channel hexagonally packed photodiode array (H-469 III, WuTech, USA). All components were assembled with precision optical positioners (Linos, Germany, Thorlabs, USA), and mounted on a high-performance vibration isolation table (BM-1, Minus-K, USA). We adjusted the optics such that each detector of the photodiode array received light from a cortical area of approximately 180 µm in diameter, and was individually amplified through a two-stage amplifier [7], with baseline subtraction. Effective bit depth of digitization approached 19 bits, at 1.6 kHz frame rate [8]. Signal amplitude is measured as millivolts of photocurrent-induced voltage.

Electrocardiograms (EKGs) were recorded with thin needle electrodes attached to each limb; electrocorticograms (ECoGs) were recorded from a silver-ball electrode, carefully placed at the margin of the imaging field. Both signals were amplified with custom-built circuitry in the range of 0.5 Hz and 450 Hz, and amplified ×1000 and ×333, respectively. These analog signals were multiplexed with the optical signals, and collected using a proprietary data acquisition script provided with the photodiode array. After recording, an EKG-triggered algorithm was used to subtract heartbeat artifact from each optical detector trace, when present [8]. For heartbeat subtraction, analysis, and data display, we used a custom library of routines for optical imaging written in Java (Sun Microsystems, USA) and Mathematica (Wolfram Research, USA), with supplements in Matlab (Mathworks, USA). In this library, peak-to-peak amplitudes and root-mean-square (RMS) noise is calculated using established methods. This library is under active development, and is freely available (http://www.sourcefourge.net/projects/nounou).

### Measurement of Resting Light Intensity

Normalization by resting light intensity (RLI) defines the intensity of voltage-sensitive dye signal as a percentage of the steady-state total fluorescence emission flux. In conventional single-chip based camera systems, the RLI is measured as the absolute baseline fluorescence, usually measured at the beginning frames of a trial. In contrast to single-chip cameras, photodiode arrays such as the device used in this report attain high signal-to-noise ratio by subtracting the steady RLI component and amplifying only the differential component of the light, prior to digitization. Therefore, in order to explicitly measure the RLI with a photodiode array, the steady total fluorescent light flux must be transformed into a variable signal. This was accomplished by briefly opening the illumination shutter. The difference obtained at the transition of non-illuminated (shutter closed) and illuminated (shutter open) conditions was measured, and taken as a measure of the RLI. Since the time constant of our optical array amplification is 1.5 s, this is essentially equivalent to a recording with DC coupling. The gain of the photodiode amplifiers was reduced during this measurement to avoid saturation. This procedure was conducted every five to ten trials during the experiment.

### Histology

At the end of an experiment, animals were deeply anesthetized with supplemental urethane and perfused transcardially with 4% paraformaldehyde in phosphate-buffered saline. Sixty micron slices were obtained with a vibratome, counterstained with DAPI (4′,6-diamino-2-phenylindole), and mounted with a polyvinyl alcohol mounting solution (MOWIOL). RH-1691 signal was imaged with a broadband red-emission filter block. Staining profiles were obtained from micrographs using ImageJ software [21].

## Results

### Functional Signal (ΔF) and Resting Light Intensity (F_RLI_) Show Different Bleaching Kinetics

We formed our initial hypothesis that total fluorescence flux (F_RLI_, commonly known as the resting light intensity) may not be an ideal reference for normalization, after noticing that F_RLI_ can show different amplitude trends and bleaching kinetics with increasing light exposure, when compared to the amplitude trends of functional signals (ΔF). Differing trends for these two measures will lead to artificial increases and/or decreases in the ΔF/F ratio, the stability of which is assumed when using the classical normalization method.

**Figure 2:**
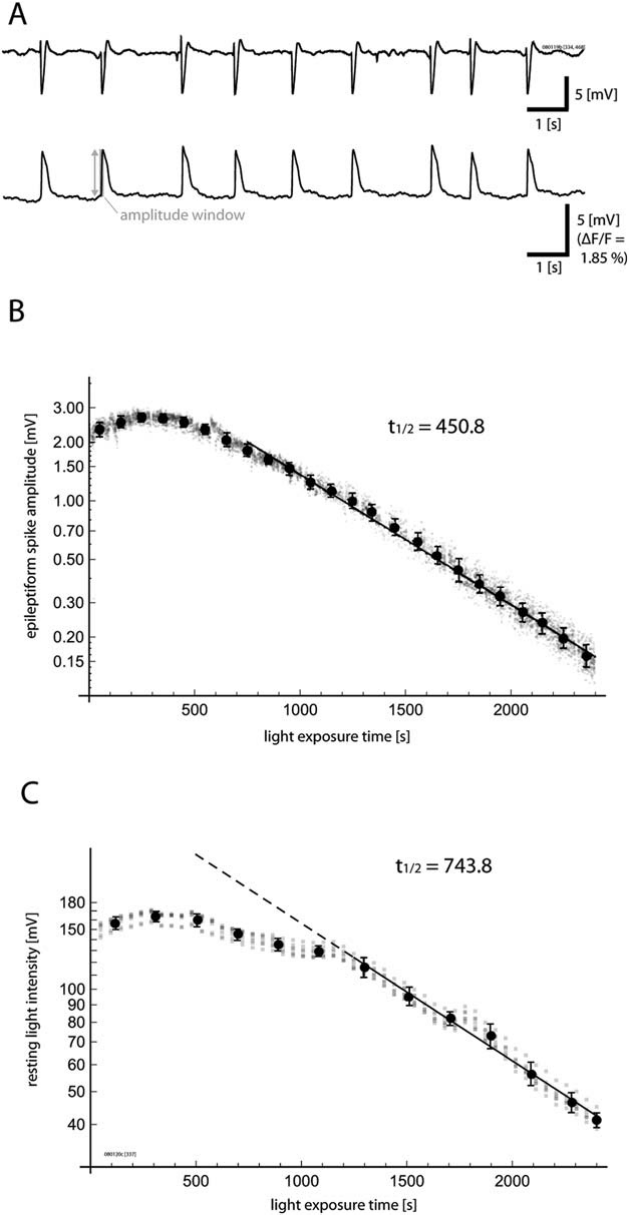
Functional signal (ΔF) and total fluorescence (F_RLI_) can show different bleaching kinetics. A. Stable, spontaneous, epileptiform spikes induced by incubation with bicuculline methiodide. The upper trace shows ECoG recording, and the lower trace shows voltage-sensitive dye fluorescence from a single detector. B. Amplitudes of photocurrent-evoked voltage from epileptiform spikes (ΔF_epileptiform_) were measured over time in a hexagonal array of seven detectors at the center of the imaging field. Each small gray dot indicates a single spike measurement from a single detector, and aggregate means and standard deviations with 100 ms bins are superimposed. At first, the signal size increases, and then gradually enters into a phase of exponential decline, This complex trend is referred to in this report as “bleaching,” with quotation marks. C. The time course of resting light intensity (F_RLI_) is displayed in the same manner. While the overall trend of this measure is similar to that of epileptiform spike amplitude with an early rise and subsequent exponential decay, the transition point between these two phases and the bleaching kinetics differ. The time constant of the later exponential phase is also slower for the resting light intensity “bleaching.” These differences in kinetics suggest a limitation of the standard ΔF/ F_RLI_ normalization.

In Figure 2, we demonstrate such instability in the relationship between ΔF and F by exploiting the high stability of epileptiform spikes elicited by bicuculline methiodide (ΔF_epileptiform_) as a functional signal source. Bicuculline methiodide can cause a stable epileptiform state in the cortex, after either local [22] or global [18], [19] application. Epileptiform spikes were detected on the ECoG, and optical spike amplitudes (ΔF_epileptiform_) were defined as the peak amplitude of optical signal within a window spanning 100 ms around the rising phase of an epileptiform spike event (Figure 2A). The amplitude of voltage-sensitive dye signal from these stable epileptiform spikes (ΔF_epileptiform_; Figure S1A, B) was plotted as a function of increasing light exposure time (Figure 2B), as reported previously [8].

While naïve theory would predict that the stochastic quantum process of bleaching should show exponential kinetics, the actual “bleaching” starts with a phase of almost stable signal amplitude (previously referred to as the “flat period” [8]), lasting for approximately 500–1000 s of light exposure depending upon conditions of staining and illumination. During this period, the size of the fluorescent signal may actually increase slightly (in this report, we refer to the overall trend as a “bleaching” curve, with quotation marks, to highlight this anomalous fact). After this initial non-exponential phase, the signal transitions to a phase of almost perfect exponential decline (previously referred to as the “declining period”).

Such complex “bleaching” of ΔF would not pose a problem for the classical normalization (ΔF/F), if F_RLI_ were to show similar kinetics as ΔF, and the ΔF/F ratio were stable over time. However, the kinetics of resting light intensity F_RLI_ (Figure 2C) often differs to a large extent from that of ΔF_epileptiform_ (Figure 2B). While F_RLI_ also shows an initial non-exponential phase and a subsequent exponential phase, the transition point of these two dynamics (the point at which the signal enters into pure exponential decay) is typically delayed, often by hundreds of seconds of light exposure. Furthermore, even after “bleaching” has reached the exponential phase, the time constant of exponential decay is invariably slower for the resting light intensity as compared to the functional signal (Figure 2C). These findings demonstrate that normalizing ΔF by F_RLI_ may introduce a bias, and also suggest that the two measures may not originate wholly from the same source within the tissue. While individual experiments varied in their exact kinetic constants, the overall biphasic “bleaching” trend and disagreement between ΔF “bleaching” and F “bleaching” were common to all experiments.

### Staining Quality Can Affect ΔF/F Ratio

One major technical challenge in voltage-sensitive dye imaging is staining of the tissue. This is especially true for complex preparations such as the *in vivo* cortex, where dye must be hydrophilic enough to permeate the cortical mantle, but must be hydrophobic enough to attach to the cell membrane and not be readily washed out [13]. Novel dyes which provide favorable compromises between these two opposing characteristics are under active development [6], [23], [24]. Since cortical staining with voltage-sensitive dye will inevitably contain a certain degree of “patchiness,” an ideal normalization method should be insensitive to the quality of staining.

**Figure 3 pone-0004041-g003:**
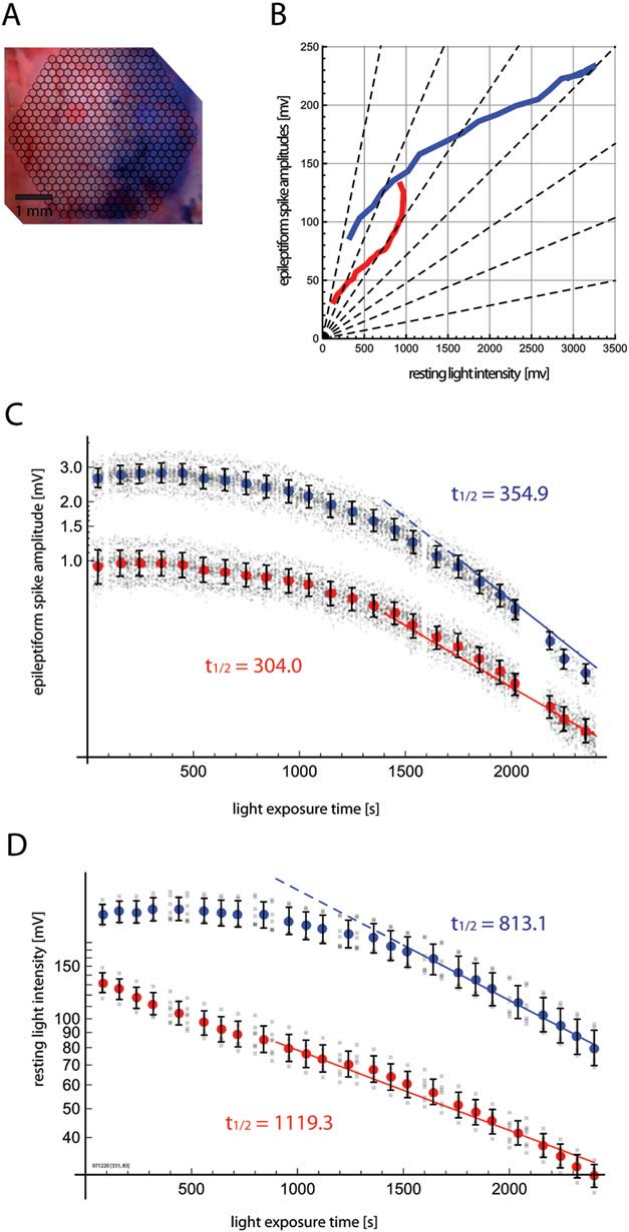
Staining quality affects ΔF and F_RLI_ differently. A. Dura in the left half of the field was protected with a small piece of tissue paper during dura permeabilization and staining. Blue and red highlights indicate the selected areas for which signal was plotted in subsequent panels. B. Epileptiform spike amplitudes (ΔF_epileptiform_) and resting light intensity (F_RLI_) were measured over time, as in Figure 2, and plotted against each other. ΔF_epileptiform_/F_RLI_ is nonlinear over the experiment. Furthermore, well-stained areas (blue) show different patterns of ΔF_epileptiform_/F_RLI_ change compared to poorly-stained areas (red). C. The timecourse of epileptiform spike amplitude (ΔF_epileptiform_) is plotted as in Figure 2B. Signal from poorly-stained areas (red) were smaller overall in amplitude, and showed somewhat faster “bleaching” kinetics within the exponential phase. D. Timecourse of resting light intensity (F_RLI_) is displayed as in Figure 2C. F_RLI_ was smaller overall in the poorly-stained half. The exponential time constant of the poorly-stained area is much slower, and a large qualitative difference in bleaching kinetics is also evident. These results demonstrate that the staining quality can affect the relationship between ΔF and F_RLI_, introducing hard-to-control biases when using the standard ΔF/F_RLI_ normalization.

In order to test whether ΔF/F can be biased by staining state, we simulated a situation where half of the imaging field is stained well with the voltage-sensitive dye, and the other half stained poorly. To realize this artificial situation, we selectively applied a previously described drying procedure [8] to the dura mater of only half of the imaging field. The other half was protected from desiccation with a small damp piece of anti-dust experimental tissue. The protective tissue was also maintained throughout staining, which prevented recirculation of dye at the dural surface in this protected area, and further reduced staining quality. This procedure resulted in a difference in staining which was visible by naked eye (Figure 3A).

Amplitudes of epileptiform spikes (ΔF_epileptiform_) and resting light intensity (F_RLI_) were measured as in the previous section. The amplitude of voltage-sensitive dye activity from epileptiform spikes (ΔF_epileptiform_) is plotted against the resting light intensity (F_RLI_) in Figure 3B, for a representative experiment. In these experiments, the relationship between resting light intensity and functional amplitude showed nonlinearities over time, straying from the linearity contours (dotted lines). Poorly-stained areas (red) have different nonlinearity trends compared to well-stained areas (blue).

Both staining conditions showed qualitatively similar “bleaching” kinetics in their functional signal (ΔF_epileptiform_)—both conditions start with an initial non-exponential phase, and subsequently converge to a similar exponential decay (Figure 3C). However, the resting light intensity (F_RLI_) trend (Figure 3D) showed large differences in their qualitative trends, between staining conditions. Whereas the well-stained areas showed bleaching in general agreement with the previous section, the poorly stained areas invariably transitioned earlier to exponential decay. In the experiment displayed, for example, the poorly-stained areas showed exponential bleaching of their F_RLI_ almost from the beginning. Since the general “bleaching” pattern of ΔF_epileptiform_ but not F_RLI_ is changed, these findings further demonstrate that staining state can alter the dynamic ratio between functional activity and resting light intensity as depicted in Figure 3B, thereby resulting in a further bias which lowers both the accuracy and precision of ΔF/F normalized values. Furthermore, the time constant of the ultimate exponential phase of F_RLI_ “bleaching” is also longer in the poorly stained region.

Taken together, these results demonstrate that staining quality can bias ΔF/F normalized values. These results also suggest that the biophysical nature of the resting light intensity may be more variable under different states of staining than the functional signal.

### Non-exponential Bleaching is Related to Illumination Intensity

In an ideal physiochemical system, dye bleaching should be a stochastic process adhering to a purely exponential decay curve. Therefore, the initial non-exponential phase of the “bleaching” curve (Figures 2, 3) defies simple explanation. Two potential sources can be hypothesized for such non-exponential “bleaching.” The first is redistribution of the dye within the biological tissue, caused either by simple diffusion or washout via cerebrospinal fluid and blood circulation. Such sources would be expected to diminish the dye signal compared to exponential, rather than increase it as is actually observed. Furthermore, such influences upon bleaching would be expected to continue throughout the experiment, instead of stabilizing, which is what we observe invariably after a certain amount of light exposure. The bleaching curve is also unaffected by pauses of up to an hour in the experiment, which further argues against dye washout being a major contributor. A second factor could be the complex three-dimensional staining profile of dye within the cortical mantle, which could lead to differential exposure to epiillumination and/or shielding of dye molecule excitation according to cytoarchitechtonic layers.

**Figure 4 pone-0004041-g004:**
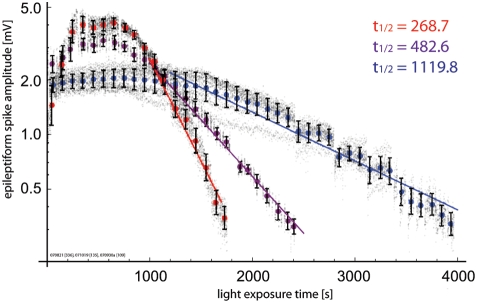
“Bleaching” kinetics are related to illumination intensity. “Bleaching” kinetics are compared from observations at three light intensities within our setup described in Figure 1: 12 V (85 W, red), 10 V (63 W, purple), and 8 V (44 W, blue). Stronger illumination is correlated with faster “bleaching” kinetics, both during the initial non-exponential phase and during the latter exponential phase.

In order to differentiate between these possibilities, we recorded “bleaching” curves at different illumination strengths. If the non-linear “bleaching” is mainly due to dye redistribution and washout, it should not be related to the illumination strength. Figure 4 displays representative “bleaching” curves from three experiments, where the preparation was recorded with varying illumination strengths. While individual “bleaching” curves can be influenced by factors such as the staining of individual preparations, in general, lower intensity illumination (blue) results in globally delayed “bleaching” kinetics, compared to higher intensity illumination (red). For the exponential phase in a well-stained preparation, an illumination voltage of 8 V on our setup (blue) results in exponential time constants of approximately 1000s, 10 V (purple) results in 350–450 s, and 12 V (red) results in time constants of under 300 s. Taken together, these findings provide further evidence to suggest that all phases of the “bleaching” curve, including the initial non-exponential phase, are directly related to physicochemical interactions of the dye and the illuminating photon flux, and not to biological or other forms of dye redistribution within the cortical mantle, which should show similar kinetics regardless of illumination strength.

### Non-exponential Bleaching is Dependent Upon Non-Neuronal Staining

The previous results strongly suggest that the non-exponential “bleaching” arises largely from illumination-related optical constraints to the simplifying assumptions required for purely exponential bleaching. One related factor which may bias the ΔF/F normalization is the non-specific staining of non-neuronal elements, for example, the dura mater.

**Figure 5:**
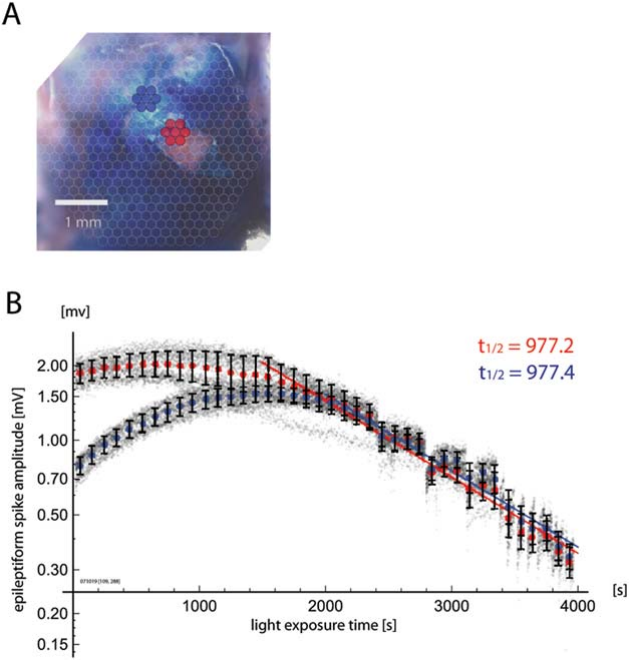
Dura mater contributes to non-exponential bleaching kinetics. A. A small area of dura was dissected and reflected. Blue and red highlights indicate the selected areas for which signal was plotted below. B. Epileptiform spike amplitudes (ΔF_epileptiform_) were measured over time, as in previous figures. Signal from areas with dura intact (blue) showed more increase in signal during the initial, non-exponential phase of “bleaching,” compared to signal from the durotomized area (red). However, in the latter, exponential phase, the exponential kinetics converged to a similar time constant and amplitude, suggesting that the dura mater contributes only to the initial, non-exponential “bleaching.” However, given the persistence of the non-exponential phase with durotomy, the dura mater cannot completely account for this phenomenon.

To explore the effects of non-specific staining on “bleaching” kinetics and ΔF/F ratio changes, we durotomized a small portion of the field after staining and recorded bleaching curves from the durotomized area and the non-durotomized area simultaneously. Figure 5 shows the results of such an experiment. Tissue in the durotomized area shows a shorter and shallower segment of non-exponential “bleaching” (red) while areas with dura intact show “bleaching” curves with more pronounced non-exponential “bleaching” (blue). After a variable non-exponential phase, however, the dura-intact areas and durotomized areas converge to exponential bleaching with a similar time constant. This final convergence is compatible with the following mechanism: at first, a significant portion of the photon flux is absorbed in the most superficial meningeal elements, including the dura. This absorption shields the underlying functional signal from sources in layer II/III, from being recorded fully. As time goes on, superficial elements with strong absorption undergo rapid bleaching, allowing the underlying functional signal to manifest, and resulting in a paradoxical increase of functional signal with “bleaching.” Once this unshielding is complete, the bleaching kinetics is relatively independent of superficial or meningeal staining. The final convergence also suggests against enzymatic and/or phototoxic complications leading to the non-exponential phase.

Given the seemingly large contribution of non-specific meningeal staining—including staining of the dura mater—to non-exponential bleaching, one may consider routine durotomy, as is commonly practiced. However, durotomy greatly decreases the mechanical stability of the cortex and hinders the recording of single-trial signal with high signal-to-noise ratio, and also leads to decreased staining efficiency in our hands, even without any visual evidence of edema or cortical damage. This decrease in yield may be due to the increased flow of cerebrospinal fluid caused by durotomy, which would dilute the dye solution. Additionally, it may also be related to inflammatory responses which can be triggered by even very careful surgery [25]. Furthermore, we find that the brain state as accessed in ECoG recordings is more stable and reproducible between animals, with dura intact preparations. Due to these effects, we find that maintenance of an intact dura during the experiment is critical for obtaining stable high-sensitivity signal with high experimental yield. Furthermore, even in the durotomized area, the general observation of non-exponential “bleaching” is still applicable (Figure 5B), and could be due to non-neuronal staining of tissue other than the dura mater.

### Dye Staining Profile Changes with Bleaching

A related non-biological factor constraining the exponential bleaching of voltage-sensitive dye signal *in vivo* is the complex staining profile of dye within the cortical mantle, and changes in this distribution. For example, if upper layers (connective tissue and other non-neuronal elements of layer I) are stained extremely well, this staining could potentially block either the penetration of illumination and/or the efflux of emitted signal from the lower layers (layer II/III), the layers which are presumably the main source of the signal [8], [26], [27]. With continued light exposure, more superficial non-neuronal elements may be bleached preferentially, contributing to the nonlinear dynamics of “bleaching.”

In order to evaluate this factor, we obtained fluorescent micrographs of the dye staining profile both before and after bleaching. In order to compare in the same animal, we prepared two identical craniotomies in the same animal, and stained them simultaneously under identical conditions. We then recorded from one craniotomy, while shielding the other with aluminum foil. The experiment exposed the cortex to a total of 2000 s of illumination at 63 W (10 V) from our tungsten-halogen lamp, with the light path as described. After this selective exposure, histological slices were prepared.

**Figure 6:**
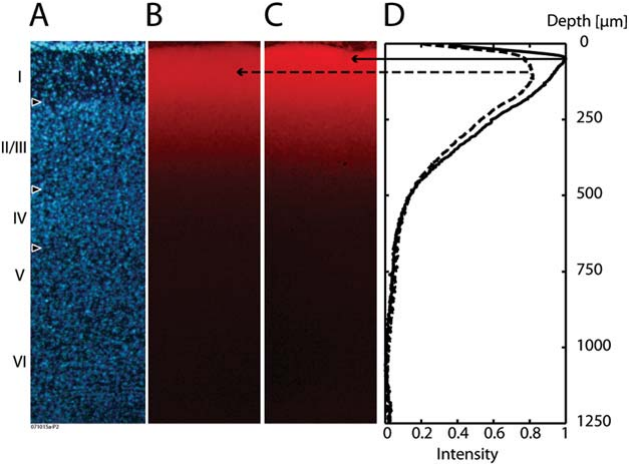
Depth profile of voltage-sensitive dye signal changes with bleaching. Stained cortex was “bleached” for 2000 seconds, with part of the cortex protected from bleaching by a piece of aluminum foil. A. Histoarchitectonic profile of the cortex with DAPI counterstain. B. Dye profile of bleached cortex. Staining with voltage-sensitive dye RH 1691 is shown. C. Dye profile of a cortical area protected from bleaching. An overall decrease in fluorescence is evident in bleached cortex (B) compared to protected cortex (C). D. Depth intensity profile of protected cortex (solid line) and bleached cortex (dotted line) are displayed, and show a slight shift in the center of the fluorescence distribution in the deeper direction, with bleaching.

Fluorescence in the bleached area is lower overall, compared to the shielded area (Figure 6B, C). However, the decrease of fluorescence due to bleaching was greater at the surface, which should cause the peak signal source under epifluorescence illumination to shift towards deeper layers, as “bleaching” progresses (Figure 6D). While this consistent change in signal source profile is not exceedingly prominent, it does involve an area closer to layers II/III, and combined with the effects described in the previous section, may help to explain the dynamic bias of ΔF/F ratio. Namely, the gradual recruitment of deeper signal sources (having more active membrane but which are relatively shielded at first), may paradoxically increase the functional signal, without causing a proportional increase in resting light intensity. Furthermore, the finding that resting light intensity can also show a signal increase with “bleaching” (Figures 2 and 3) suggests that the dye molecules staining meningeal and superficial tissue elements may have a physical microenvironment leading to fluorescent emission with lower efficiency.

### Non-epileptiform Functional Signal Bleaches Similarly to Epileptiform Signal

The final question we asked was whether other forms of functional neural activity, such as “spontaneous” sleep-like slow waves [8], [17], [28], would also show similar biophysical bleaching as the epileptiform signal. If dye signal from sleep-like signals shows bleaching kinetics distinct from epileptiform signal, this would imply that the biophysical sources of these two signals are distinct, and that normalizing with the epileptiform signal would entail a new set of problems distinct from the problems associated with ΔF/F.

**Figure 7:**
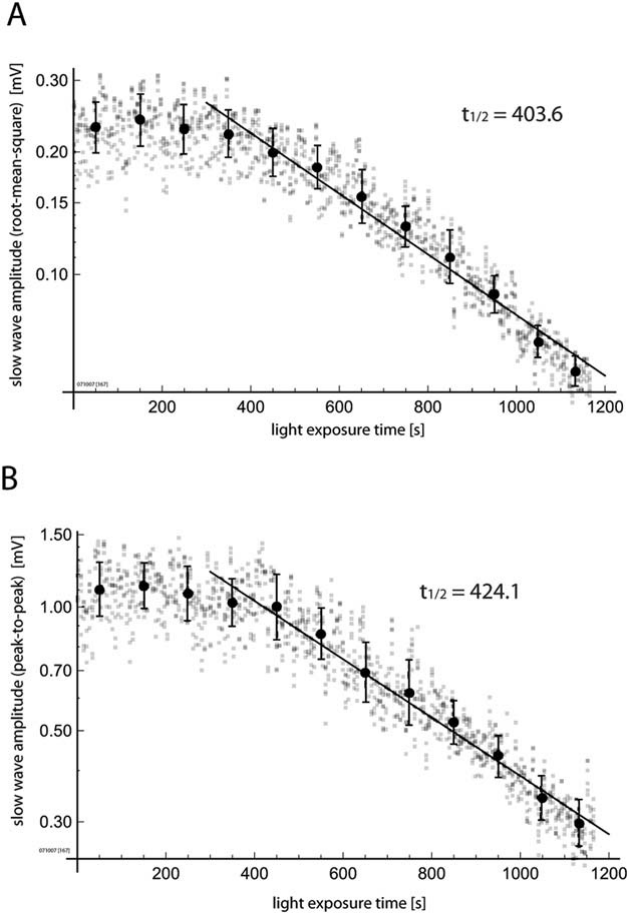
Non-epileptiform functional signal shows similar “bleaching” as epileptiform signal. The “bleaching” of spontaneous sleep-like slow wave activity was accessed under stable isoflurane anesthesia. A. Root-mean-square power of the slow wave activity. B. Peak-to-peak amplitude of the slow wave activity. Both measures show “bleaching” kinetics similar to those observed with epileptiform activity (ΔF_epileptiform_).

In order to evaluate the bleaching of sleep-like waves and non-epileptiform functional activity, we recorded from stably anesthetized preparations with 0.8% isoflurane, without application of bicuculline methiodide (all other experiments presented were done under IP urethane anesthesia). Under this condition, slow sleep-like waves of spontaneous activity can be observed [8]. By maintaining careful physiological support of the animal, including maintenance of normothermia, normocapnia and lung state (with careful conditioning of inspiratory gas), the sleep-like wave frequency profile of both ECoG and voltage-sensitive dye activity of the animal can be kept stable for hours. We measured the amplitude of these “spontaneous” sleep-like waves, as the preparation was exposed to fixed durations of illumination. The “bleaching” curve of this activity is displayed in Figures 7A and 7B. Both the root-mean-square (RMS) amplitudes (Figure 7A) and peak-to-peak amplitudes (Figure 7B) show patterns comparable to “bleaching” of epileptiform signals displayed in Figures 2–5. Under the minimal assumption that the cortical state of anesthesia-related “spontaneous” activity was stable over time in our experiments (Figure S1C), these results demonstrate that the biophysical substrates of such functional activity “bleach” with similar kinetics as the epileptiform spike amplitude, and therefore, imply that the epileptiform spike amplitudes may provide an appropriate basis for their normalization.

### Demonstration of Functional Normalization

**Figure 8:**
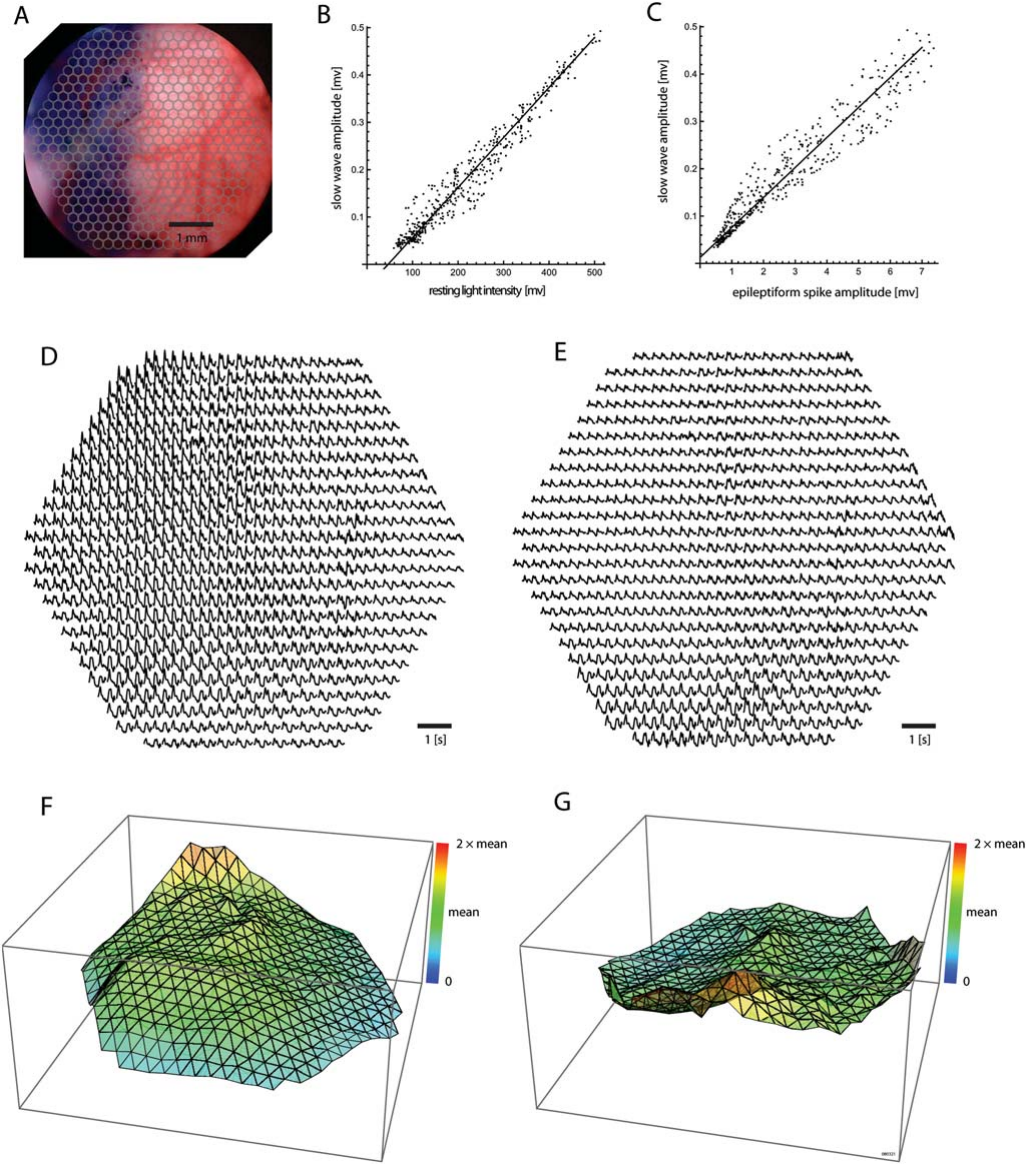
Normalization with resting light intensity (ΔF/F_RLI_) compared to functional normalization with epileptiform spike amplitude (ΔF/ΔF_epileptiform_). A. Half of the cortex was stained well, as in Figure 3. B. Raster plot of sleep-like slow wave activity and resting light intensities from all 464 detectors in the imaging field. A zero-point shift is evident, which demonstrates a nonlinear relationship between ΔF and F_RLI_ at this timepoint. C. Raster plot of sleep-like slow wave activity and epileptiform spike amplitudes after global disinhibition with bicuculline methiodide, from each of 464 detectors. The ΔF/ΔF_epileptiform_ ratio is linear. D. Sleep-like slow wave activity from an imaging area around V1, as normalized with resting light intensity (ΔF/F_RLI_). E. The same signal, normalized with epileptiform spike amplitude (ΔF/ΔF_epileptiform_). More inhomogeneity is evident with ΔF/F_RLI_ normalization. F. The root-mean-square of sleep-like slow wave activity under stable isoflurane anesthesia is displayed, normalized by the resting light intensity (ΔF/F_RLI_). The z-axis is set from zero to two times the mean normalized value over the field, in this and the next panel. This common z-axis scaling allows comparison of field homogeneity between different normalized value arrays. G. The same root-mean-square power normalized by the amplitude of spontaneous epileptiform spikes induced by application of bicuculline methiodide (ΔF/ΔF_epileptiform_). The power normalized with epileptiform acitivity is more homogeneous throughout the field.

In order to demonstrate that the epileptiform signal amplitude (ΔF_epileptiform_) can indeed provide a useful normalization of other functional signals, we display the spatial distribution of sleep-like activity across the imaging field, in a preparation where only half of the cortex was stained well, as in Figure 3 (Figure 8A).

Figures 8B and 8C plot the root-mean-squared power of sleep-like slow wave amplitude (ΔF_SW_) in all 464 detectors of our imaging field, against either the resting light intensity amplitudes (F_RLI_; Figure 8B) or against the epileptiform spike amplitudes (ΔF_epileptiform_; Figure 8C) measured at these same detector locations. The correlation between ΔF_SW_ vs. F_RLI_ shows a zero-point shift on the abscissa (Figure 8B), which could potentially be explained by non-neuronal staining—non-neuronal staining would contribute to the resting light intensity, but not to functional signal. Such a zero-point shift bias is not prominent in the correlation between ΔF_SW_ and ΔF_epileptiform_ (Figure 8C). This bias in the ΔF_SW_/F_RLI_ linearity, observed at a single time point, would be expected to further change dynamically over time.

To further demonstrate the effects of functional normalization, we display the spatial distribution of this same data signal with either the classical ΔF/F_RLI_ normalization (Figure 8D) or with the functional normalization ΔF/ΔF_epileptiform_ (Figure 8E), using a “page” plot of a representative segment of sleep-like slow wave activity—traces are plotted at locations corresponding to their geometric layout. These plots demonstrate that the functional normalization ΔF/ΔF_epileptiform_ (Figure 8E) provides a more homogeneous activity measure across the cortex, compared to the classical ΔF/F_RLI_ normalization. The same data is plotted as three-dimensional surface plots, to underline the higher homogeneity obtained by functional normalization (Figure 8G) compared to ΔF/F_RLI_ normalization (Figure 8F). Therefore, to the extent that sleep-like synchronized activity is common throughout the various cortical fields [29], these findings demonstrate that functional normalization may, to the first approximation, mitigate biases inherent in the classical ΔF/F_RLI_ normalization.

## Discussion

Voltage-sensitive dye imaging [30] has evolved into a premiere method for observing patterns of population mass action in the intact mammalian cortex [1]. Relatively inexpensive apparati can now be combined to provide reliable, single-trial recordings with high temporal resolution and signal-to-noise ratio [8]. This maturation will open a new vista upon the physiology of network activity patterns [1], [12], [14], [15], [28], [31], [32], [33]. However, the increasing application of this method also warrants a review of the nature of the population signal, and methods of quantification. For example, it is well known that complex variables such as membrane area or topology of measurement, nonspecific fluorescence from dye molecules, situated outside of active membrane, and sites of dye binding within the cell can influence the optical measurement of voltage-sensitive dye signal [34], In mammalian preparations *in vivo*, it has been previously conjectured that the ΔF/F measure can contain biases, and that functional normalization such as with epileptiform activity has the potential to provide a more stable basis for normalization [35]. However, conjectures such as these have not been systematically explored previously.

Although voltage-sensitive dye signals in cortex *in vivo* originate mainly from superficial layers and show strong correlations to dendritic post-synaptic potentials under certain conditions [27], it is difficult to routinely and comprehensively localize the source of dynamic patterns of mass action *in vivo*, and indeed, sub-populations of the cortical mantle may contribute differently under varying cortical states [36]. However, it is clear that the activity stems mainly from some element of cortical activity in all cortical states—surface application of the GABA_A_ agonist muscimol (1 µg/µl) rapidly and invariably eliminates all types of population activity observed, including epileptiform activity, spontaneous sleep-like activity under lighter anesthesia, burst-suppression activity under deeper anesthesia, and sensory-evoked activity under deep and light anesthethesia—this elimination of activity occurs within a minute of muscimol application (data not shown).

The normalization measure that we propose here takes advantage of the “hypersynchronized” nature of epileptiform spikes. This type of activity allows recording of the voltage-sensitive dye signal corresponding to near-simultaneous activation of a majority of the recorded neural elements in the underlying cortical population, and therefore may provide an ideal basis for functional normalization. However, the induction of epileptiform spikes with bicuculline methiodide is irreversible within an acute experiment [19], and therefore, this normalization basis cannot be repeatedly measured between physiological recording trials. One strategy to circumvent this issue is to limit recording to the early, non-exponential phase of the bleaching curve where the “bleaching” is less significant, and a single normalization basis may prove sufficient [8]. In the setup described in this manuscript, this is equivalent to a total exposure time of 500 s per experiment, which allows sufficient data to be accumulated given a careful experimental plan. When longer durations of data must be gathered, the amplitude of stable spontaneous activity patterns, such as in synchronized cortical activity under anesthesia, may potentially serve as an alternative normalization source (Figure 7)—albeit with lower signal-to-noise ratio.

Functional normalization is particularly important for maintaining accuracy when comparing across experiments, since as we have shown, staining quality can greatly affect the ΔF/F ratio (Figure 3). It should also improve the comparison of population activity across cytoarchitectonic areas within the same experiment [15], [28], since various areas have different tissue compositions, which may confound ΔF/F through its effects on staining quality. For example, in our experience, topographical alignment and perfusion patterns of various areas can alter staining quality greatly—for example, staining is invariably much less robust in rodent auditory cortex, where curvature is higher, and perfusion through the middle cerebral artery is more noticeable than in visual or somatosensory areas. Such anatomically fixed confounding factors for the ΔF/F have the potential to cause inaccuracies in experimental results, even when precision is relatively high.

The potential utility of functional normalization extends to optical methods beyond voltage-sensitive dye recording. For instance, in the recently proposed quantum-dot based voltage recording [37], bleaching should not be a problem, but non-specific staining can be expected to remain. This non-specific staining is unlikely to have a constant ratio to neuronal staining in a complex tissue such as the cortex, and therefore, functional normalization may provide a more accurate normalized measure compared to ΔF/F. Even when non-specific staining can be better distinguished, such as when recording calcium dynamics with multi-photon laser scanning microscopy, precise calibration of intracellular calcium levels are required to make inter-experiment comparisons. Normalization with a stable source of stereotypical activity, such as that induced by bicuculline methiodide, may allow an easy confirmation of such painstaking calibrations.

Given the complexities inherent in normalization of voltage-sensitive dye imaging signal, a further implication of these findings is that normalization-independent metrics such as waveform propagation patterns[12], [14], [16], oscillations in single detectors[38], and phase coupling between detector pairs[39] may better characterize the biological information which is obtained by high-sensitivity imaging.

In summary, the resting light intensity (RLI) originates to a large extent from non-excitable tissue, and therefore, when used as a source for normalization, can cause biases that are dependent upon depth distribution of the dye, extent of bleaching, staining quality and dye concentration. Therefore, when accurate quantification of population activity in the cortex is desired, such normalization is less than ideal. Global application of bicuculline methiodide causes “hypersynchronization” of cortical networks in spontaneous bursts of epileptiform activity, which give an index of voltage-sensitive dye signal amplitude at near-maximal neural activity. Normalizing from this reference allows a more accurate quantification of neuronal activation, which is less biased by differences in staining. Other forms of stable functional activity, such as sleep-like slow wave activity, may also potentially be used as a normalization reference. These functional normalizations allow more accurate quantification of the amplitude of population activity within complex neuronal populations. They further suggest that normalization-independent metrics may better capture the essence of the biological information which is obtained by high-sensitivity imaging.

## Supporting Information

Figure S1 Electrical stability of epileptiform spikes and sleep-like slow waves. A,B. Epileptiform spike electrocorticograms recorded at an early point in the experiment (A) and 2 hours later (B). The amplitude and waveforms of the spikes can be maintained stably. C. Sleep-like slow waves recorded under low isoflurane anesthesia (0.8%), recorded over 2.5 hours. The amplitude and waveforms of activity can be maintained stably.
(11.05 MB PDF)
