# Supplementary figures and images for: Normalization of Voltage-Sensitive Dye Signal with Functional Activity Measures
Source: PLoS One. 2008 Dec 24;3(12):e4041. doi: 10.1371/journal.pone.0004041 (PMC2612132; doi:10.1371/journal.pone.0004041)

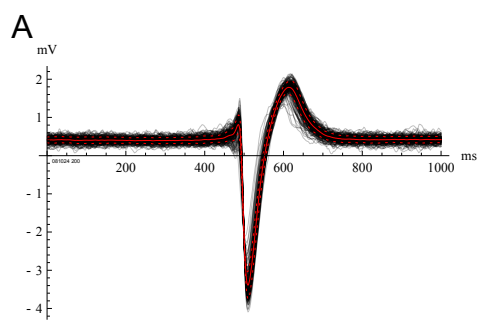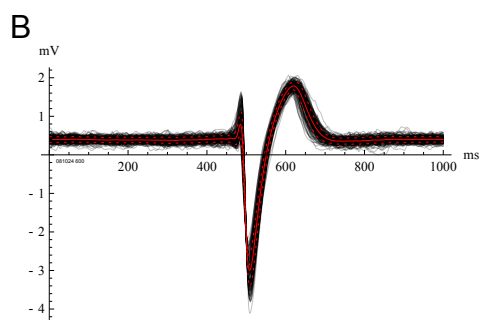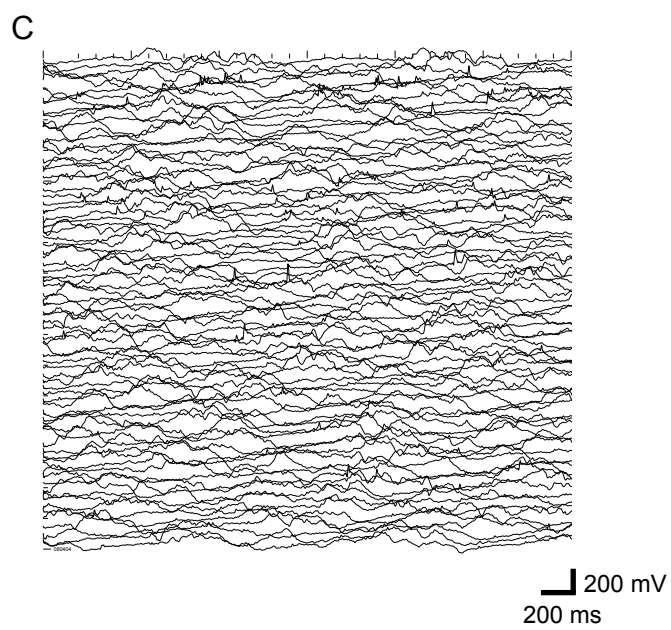

Supplement: Figure S1 — Electrical stability of epileptiform spikes and sleep-like slow waves. A,B. Epileptiform spike electrocorticograms recorded at an early point in the experiment (A) and 2 hours later (B). The amplitude and waveforms of the spikes can be maintained stably. C. Sleep-like slow waves recorded under low isoflurane anesthesia (0.8%), recorded over 2.5 hours. The amplitude and waveforms of activity can be maintained stably. (11.05 MB PDF) [file pone.0004041.s001.pdf]
